# *Announcement*: Community Preventive Services Task Force Recommends Interventions to Increase Healthier Foods and Beverages in Schools

**DOI:** 10.15585/mmwr.mm6616a7

**Published:** 2017-04-28

The Community Preventive Services Task Force recently posted new information about four findings on its website: 1) Obesity: Meal and Fruit and Vegetable Snack Interventions to Increase Healthier Foods and Beverages Provided by Schools*; 2) Obesity: Supporting Healthier Snack Foods and Beverages Sold or Offered as Rewards in Schools^†^; 3) Obesity: Multicomponent Interventions to Increase Availability of Healthier Foods and Beverages in Schools^§^; and 4) Obesity: Increasing Water Access in Schools.^ ¶^

Established in 1996 by the U.S. Department of Health and Human Services, the task force is an independent, nonfederal panel of public health and prevention experts whose members are appointed by the director of CDC. The task force provides information for a wide range of persons who make decisions about programs, services, and other interventions to improve population health. Although CDC provides administrative, scientific, and technical support for the task force, the recommendations developed are those of the task force and do not undergo review or approval by CDC.

